# Supplementary material for: Association between oral microbiome and seven types of cancers in East Asian population: a two-sample Mendelian randomization analysis
Source: Front Mol Biosci. 2023 Nov 21;10:1327893. doi: 10.3389/fmolb.2023.1327893 (PMC10702768; doi:10.3389/fmolb.2023.1327893)
Supplement: Supplementary file 1 [file DataSheet1.ZIP › Supplementary Materials/MR plots of saliva/saliva═╝/colorectal cancer/pheno.2599_to_colorectal cancer_leave_one_out.pdf]

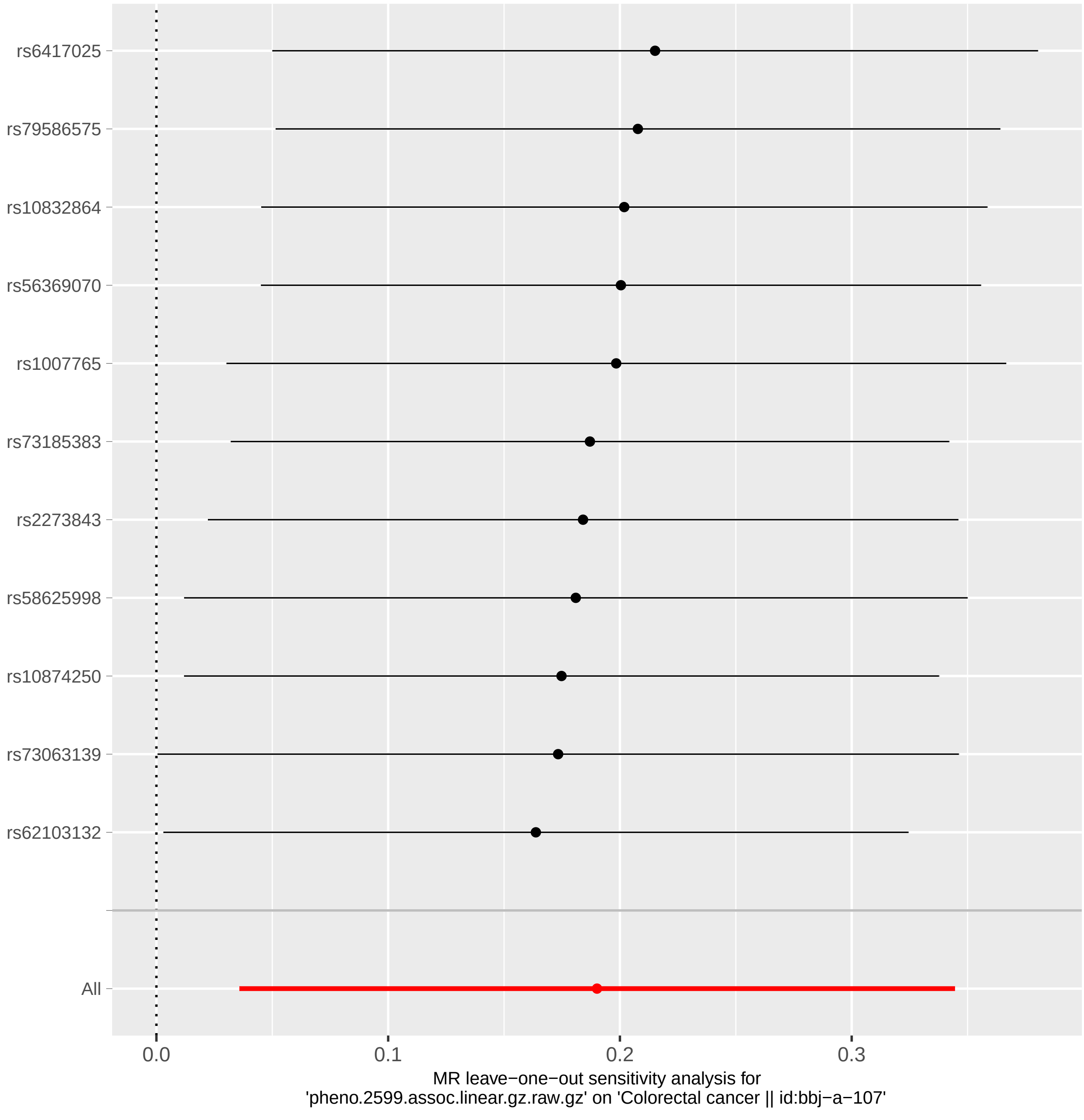

rs6417025
rs79586575
rs10832864
rs56369070
rs1007765
rs73185383
rs2273843
rs58625998
rs10874250
rs73063139
rs62103132
All
0.0
0.1
0.2
0.3
MR leave−one−out sensitivity analysis for
'pheno.2599.assoc.linear.gz.raw.gz' on 'Colorectal cancer || id:bbj−a−107'
